# Detailed Experiment-Theory Comparison of Mid-Infrared Metasurface Perfect Absorbers

**DOI:** 10.3390/mi11040409

**Published:** 2020-04-14

**Authors:** Naoki To, Saulius Juodkazis, Yoshiaki Nishijima

**Affiliations:** 1 Department of Electrical and Computer Engineering, Graduate School of Engineering, Yokohama National University, 79-5 Tokiwadai, Hodogaya-ku, Yokohama 240-8501, Japan; to-naoki-jp@ynu.jp; 2 Optical Sciences Centre and ARC Training Centre in Surface Engineering for Advanced Materials (SEAM), School of Science, Swinburne University of Technology, Hawthorn, VIC 3122, Australia; sjuodkazis@swin.edu.au; 3 Institute of Advanced Sciences (IAS), Yokohama National University, 79-5 Tokiwadai, Hodogaya-ku, Yokohama 240-8501, Japan; 4 Tokyo Tech World Research Hub Initiative (WRHI), School of Materials and Chemical Technology, Tokyo Institute of Technology, 2-12-1, Ookayama, Meguro-ku, Tokyo 152-8550, Japan

**Keywords:** metasurface, mid infrared absorption, perfect absorption

## Abstract

Realisation of a perfect absorber A=1 with transmittance and reflectance T=R=0 by a thin metasurface is one of the hot topics in recent nanophotonics prompted by energy harvesting and sensor applications (A+R+T=1 is the energy conservation). Here we tested the optical properties of over 400 structures of metal–insulator–metal (MIM) metasurfaces for a range of variation in thickness of insulator, diameter of a disc and intra-disc distance both experimentally and numerically. Conditions of a near perfect absorption A>95% with simultaneously occurring anti-reflection property (R<5%) was experimentally determined. Differences between the bulk vs. nano-thin film properties at mid-IR of the used materials can be of interest for plasmonic multi-metal alloys and high entropy metals.

## 1. Introduction

**Figure 1:**
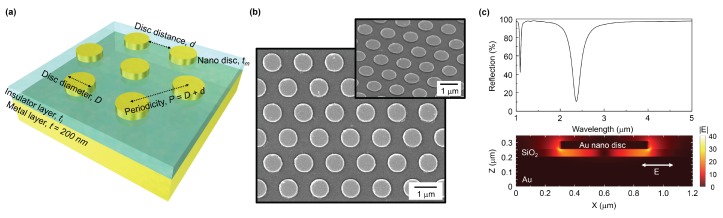
(**a**) Schematics of the metal–insulator–metal (MIM) metasurface with definition of the geometrical parameters. (**b**) SEM image of the fabricated metasurface; inset shows a 45${}^{\circ }$-tilted view SEM image. (**c**) Finite-difference time-domain (FDTD) simulated reflection spectrum of metasurface with a disc diameter of 300 nm, period of 1200 nm, SiO${}_{2}$ thickness of 50 nm. A cross sectional image of electric field under excitation of x-polarized light reveals a dipole nature of distribution.

Metal–insulator–metal (MIM) type of metasurfaces [1] are used to maximise the absorbance *A* with no transmittance *T* and reflectance *R* at the resonance. For the mid-infrared wavelengths, MIM metasurfaces (Figure 1) could be designed as photo–thermal energy converters which convert luminous IR light into heat [2,3,4]. Broadband mid-IR absorbers were demonstrated by a metal-coated polystyrene micro-bead colloidal lithography, which also have a wide-angle performance [5]. The MIM metasurfaces with metallic nano-/micro-structures on the top surface require only one lithography step [1]. They can provide a versatile control of intensity, phase, polarisation of the reflected, scattered, transmitted light and can be easily designed for a required wavelength by choice of different shape/material resonators [6]. Nano-disk MIM absorbers using lossy Ti nano-disks (instead of the usual plasmonic Au) showed high A>90% absorbance at visible near-IR spectral window [7]. An analytical expression of the relectance coefficient was derived and showed that minimisation of reflectance is achieved by control of the intrinsic and radiative losses [8]. The ratio of the quality factors of absorption $Q_{a}$ and radiation $Q_{r}$ losses define the minimum of the achievable reflectivity $r=\left(Q_{a}/Q_{r}-1\right)/\left(Q_{a}/Q_{r}+1\right)$, hence the maximum absorbance achievable by MIM structures at $Q_{a}/Q_{r}\to 1$ [9]. With a MIM reflector placed on a nano-thin and flexible Si_3_N_4_-membrane with an electrically biased graphene layer, spectral tunability of the resonance wavelength of the perfect absorber at mid-IR wavelengths was recently demonstrated [9]. Simple nano-disk MIM surfaces (Figure 1) showed a near perfect absprbance A=99% at 1.6 μm wavelengths with high refractive index sensitivity close to the resonance [10]. The Drude model is commonly used to model perfect absorbers at near-IR wavelengths [11], where the antenna S-parameters $S_{11}\equiv R$ and $S_{21}\equiv T$ define the impedance matching $Z=\sqrt{\mu }/\sqrt{\varepsilon }=1$ (to the air at the input port) for the resonance wavelength when the permittivity and permeability are equal ε=μ. The resistive heating can readily be calculated as $Q\left(\omega \right)=\varepsilon_{0}\omega Im\left[\varepsilon_{M}\left(\omega \right)\right]{\left|E\left(\omega \right)\right|}^{2}/2$, where $\varepsilon_{0,M}$ are permittivities of the free space and metal, respectively, and E(ω) is the E-field of light [11].

In our previous work, we have demonstrated the effect of metal and insulator patterns as absorbers and thermal emitters [12]. Heat generation using metasurfaces can provide a mid-IR wavelength source, detectors with narrow bandwidth, and radiative cooling at the atmospheric window wavelengths 3–7 μm (T≈90%) and 8–13 μm (T≈80%−60%). Predictive models of MIMs and other metamaterials with required optical and thermal properties have to be designed based on numerical modeling and optimisation. In this regard the finite-difference time-domain (FDTD) method based on the exact solution of Maxwell’s equations is expected to provide the quantitative match to the performance of experimental structures provided material property (electric permitivity ε) is known at the used wavelengths. However, we showed recently that the experimentally made structures have different optical responses from the simulated ones due to settle differences in interface roughness and geometry of the nano-pattern [13]. This study is dedicated to address the issue that the maximum absorption A→1 is not easily engineered by MIM structures with simultaneously occurring zero reflection condition R→0. It is very challenging to realise the perfect absorption condition A→1 [13]. A wide parametric study of geometrical characteristics of the MIM patterns is presented to reveal their effect on the optical response of MIM patterns (over 400 samples). In this work we extend the parameter space of metasurfaces and systematically study dependence of *R* and *A* on the disc diameters *D*, intra disc separation distance *d* (the period is P=D+d), insulator thicknesses $t_{i}$ in the case of fixed MIM materials: Au film, SiO${}_{2}$, Au nanodisc. Optimisation towards A=1 while R=0 can be guided by tendencies revealed from the experimental data. All samples (400+) were made during the same material deposition run. This approach inherently takes into account specific properties of thin insulator, metal films and their interfaces dependent on the deposition method, in this case, electron beam (EB) evaporation.

## 2. Experiments

### 2.1. Fabrication and Characterization of MIM Metasurfaces

Metasurfaces were fabricated with conventional electron beam lithography (EBL) [14]. A 200-nm-thick film of Au was deposited on the glass substrate with a 5 nm Ti adhesion layer by EB evaporation. Then, a SiO_2_ insulator layer was deposited over it with thickness varying from 30 to 100 nm with 10 nm increments. After spin coating the EB resist (a twice diluted ZEP 520 A), EBL was implemented to draw a pattern of circular disks with hexagonal lattice arrangement. Diameter of the disk *D* was set from 400 to 800 nm in 100 nm steps while intra-disc distance *d* was changed from 100 to 1000 nm. After development of EBL-defined disk patterns, a 5 nm layer of Ti followed by 50 nm thickness of Au were deposited. The final step of lift-off in a heated solution of dichloromethane, acetone and methanol was then carried out to obtain the pattern of nano-discs on the top surface.

All the structures were drawn on the same substrate to avoid heterogeneity of the samples (400+) due to fluctuation of the thickness of the first Au film, insulator layer and that of Au nano-discs. This was an essential feature of this study which allowed to compare the influence of purely geometrical factors of MIM metasurfaces. The reflection spectra of metasurfaces were measured with a micro-spectroscopic Fourier transform FT-IR setup. A R∼98% reflection Au mirror was used as a reference for the reflection spectra. Also we have confirmed a zero transmission with the same FT-IR system. Illustration of the sample geometry and scanning electron microscopy (SEM) images are shown in Figure 1.

### 2.2. FDTD Simulations

The finite-difference time-domain (FDTD) simulations were performed with commercially available software (FDTD solution, Lumerical, Inc.) with three nodes of in-home assembled workstations. The area of simulations was set to 2.5 periods in the x-direction and 2.0 periods in the y-direction for the x-polarised plane wave light source of unit intensity ${\left|E\right|}^{2}=1$. The dielectric material properties, permittivity $\sqrt{\varepsilon }=n+ik$, where n+ik is the complex refractive index were taken from the Lumerical database (Palik’s spectroscopic reference data [15]) for the mid-IR wavelength region as the default setting.

## 3. Results and Discussions

### 3.1. Comparison of Experimental and Numerical Optical Properties

**Figure 2:**
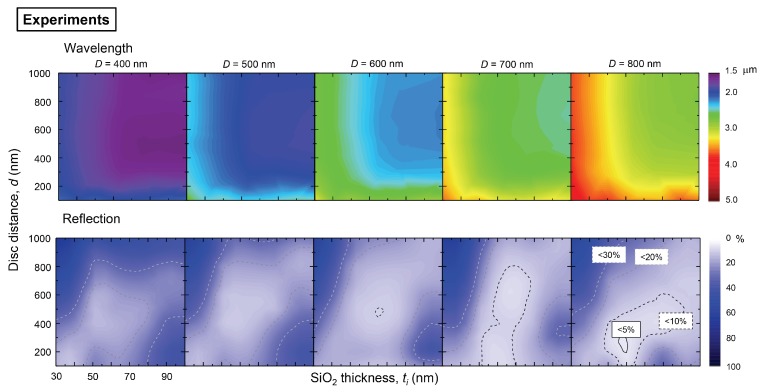
Experimental results of the resonance wavelength (color-coded) and minimum reflectance *R* obtained from series of reflection spectral measurements (400+) with parameters of disc diameter *D*, intra-disc separation distance *d* and SiO_2_ thickness $t_{i}$. Number of samples measured for one *D* was (10×8)=80 for different $t_{i}\times d$.

Figure 2 shows experimental results of the resonance wavelength and reflectance at its minimum. The wavelength of the reflectance *R* minimum is strongly affected by the disc diameter *D* and SiO${}_{2}$ thickness, but it is almost insensitive to the intra-disc separation when it is larger than 200 nm. When the SiO${}_{2}$ layer becomes thicker, the wavelength of the minimum *R* is blue-shifted and becomes constant after the specific threshold thickness. The 40 nm is the characteristic thickness of SiO${}_{2}$ for the D=400 nm diameter, but 60 nm for the D=800 nm. These tendencies are similar for all disc diameters.

There are two main interactions in nano-disc patterns. First, the interaction of the disk with the metal film. For a thinner SiO${}_{2}$ film, the induced mirror-dipole in the metal film is coupled with the dipole of the plasmon resonance. The second interaction is the dipole–dipole interaction between the closest nano-disks. As these dipole–dipole interactions become stronger, the resonance peak wavelength red-shifts. Of course there is another factor, i.e., a periodic light scattering occurs and affects the plasmon resonance wavelength. The electric field extending from the metal surface into air is strongly wavelength dependent, at a longer wavelength a longer evanescent field extension is observed. Therefore the larger diameter disks, which resonate at the longer wavelengths, have a larger threshold for the separation distance and thickness of insulator layer as compared to the smaller diameters.

These interactions between nano-discs and film as well as with the neighboring discs also affect the reflectance. When the intra-disc distance becomes smaller, the reflectance starts decreasing (an increase in optical losses) due to the increased density of nanodiscs (a larger surface area covered by metal). However, the interaction between nano-discs generates a de-localization of the electric field, resulting in an increase of the reflectance (a decrease of absorption). This tendency is much clearer in the thick SiO${}_{2}$ layers, which have less interaction between mirror dipoles. In the case of thinner SiO${}_{2}$, reflectance still decreases until 100 nm of intra-disc separation distance *d*. Therefore, the color map in Figure 2 shows the tendency of *R* to increase towards the larger SiO_2_ thickness.

The total mirror dipole interaction can be described by the quadrupole mode that causes a strong electric field localization and larger absorption in the insulator layer. In the series of experimental studies, we found less than the 5% reflectance condition for the D=800 nm discs, $t_{i}=60$ nm SiO${}_{2}$ and 300 nm intra-disc separation distance.

**Figure 3:**
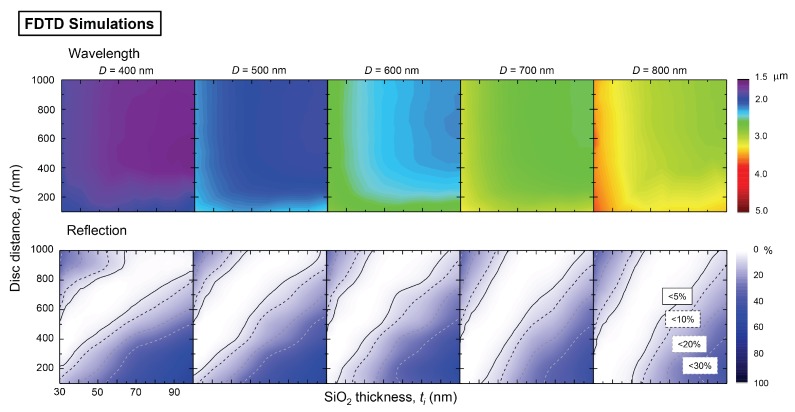
FDTD simulation results of resonance wavelength (color-coded) and minimum reflectance that were obtained from the series of reflection spectral measurements with parameters of disc diameter *D*, disc distance *d*, SiO${}_{2}$ thickness $t_{i}$. See Figure 2 for comparison with experimental results.

Figure 3 shows the FDTD simulation results for the same geometry of patterns as in the experimental study of reflectivity. Similar qualitative tendencies of the resonance wavelength are reproduced as in the experiments. However, the reflectance values obtained by FDTD simulations are quite different from the experimental results. Usually, the FDTD simulations show a lower reflectivity at the minimum.

### 3.2. Effects of Metal Permittivity

**Figure 4:**
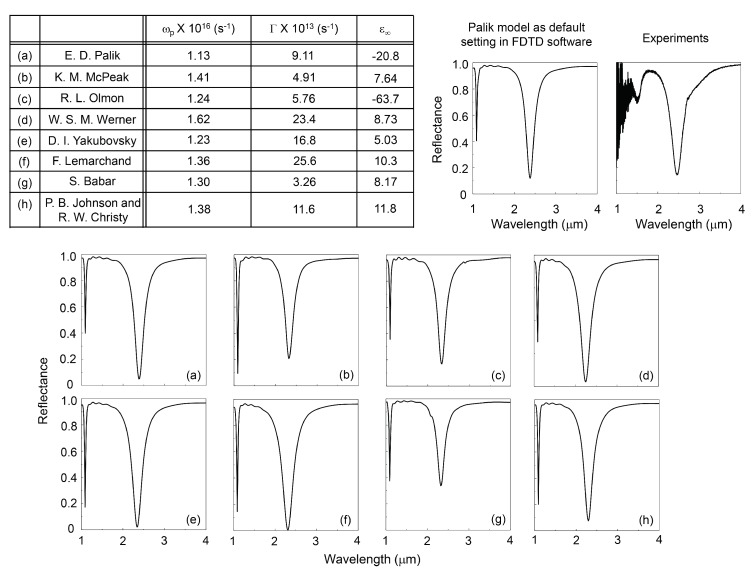
Drude parameters of the permittivity of Au were obtained from the references listed below. The parameters of D= 600 nm, P= 1200 nm, $t_{i}$ = 50 nm were used: (**a**) Palik [15], (**b**) McPeak [18], (**c**) Olmon [19], (**d**) Werner [20], (**e**) Yakubovsky [21], (**f**) Lemarchand [22], (**g**) Babar [23] and (**h**) Johnson and Christy [16]. The FDTD simulation results by (a) Palik parameters from the FDTD Lumerical software database (a default) were used for the Drude model calculations (see table). Also, the experimental reflection spectrum is shown.

Typically, a commercial FDTD software includes a database library of the electric susceptibility (refractive index) for simulations at a specific wavelength range. The used FDTD Lumerical software has a list of popular reference data including Palik [15], Johnson and Christy [16], and CRC [17] (C. G. Olson et al., unpublished data, etc.) among others in the database library of dielectric parameters. It is well known that the susceptibility tends to show different values according to the sample fabrication methods, thickness of the film and analytical methods of characterisation. In Figure 4, we listed several conventional dielectric parameters from popular data sets by Palik, Mcpeak, Olmon, Werner, Yakbousky, Lermarchand, Johnson and Christy [15,16,18,19,20,21,22]. These parameters show variation in wavelength and spectral shape. Therefore, we analyzed permittivity using the Drude model at longer than 800 nm wavelength, where it is less affected by the Lorenz part of the permittivity. The Drude equation was used:

$$\varepsilon \left(\omega \right)=\varepsilon_{\infty }-\frac{\omega_{p}^{2}}{\omega^{2}+\Gamma^{2}}+i\frac{\omega_{p}^{2}\Gamma }{\omega (\omega^{2}+\Gamma^{2})}$$

where $\varepsilon_{\infty }$ is the permittivity at high frequency region, $\omega_{p}$ is the plasma frequency that is related to the free electron density and effective mass of the electron, $\omega_{p}=\sqrt{N_{e}^{2}/\varepsilon_{0}m}$, Γ is the damping constant of the free electron oscillation, which is the inverse of the relaxation time τ. Using these three parameters as free variables, the permittivity was fitted with the least-square method. The result is shown in the Table section of Figure 4. The $\omega_{p}$ varies only from 1.13 to 1.62 $\times 10^{16}$ s${}^{-1}$ for different models. However, the Γ values are spread over a wide range from 3.26 to 25.6 $\times 10^{13}$ s${}^{-1}$.

The $\varepsilon_{\infty }$ is the extrapolated value from the lower frequency region. Therefore, the data have a large fluctuation but it has a minor influence (∼ 1%) for the real part of permittivity at the mid-IR spectral region. Using these values in the range from 1 to 10 μm, the values of permittivity were imported into the material database for FDTD simulations (Lumerical). As a default, the Palik model was used in the simulations with results shown in Figure 2 and Figure 3. The internally fixed parameters (Lumerical) of the tolerance and maximum order coefficients were set to 0.1 and 6, respectively. Tolerance specifies the target root-mean-square (rms) error between the experimental data and the calculated model. Maximum order coefficients set the maximum number of coefficients allowed in the model. It was found that these values are not completely compatible with the imported permittivity. Due to this difference of the internal parameters, the FDTD simulation results were slightly different from the Drude modeled parameters shown in Figure 4a. We set them to 0.01 and 10 to obtain well fitted data within a practically justifiable calculation time. The FDTD simulation results with the structural parameters of 600 nm diameter disks, 1.2 μm period, and 50 nm of SiO${}_{2}$ thickness were simulated with all nine engineered permittivities. As a reference, we also calculated data according to the Palik model with default settings in Lumerical. The results are shown in Figure 4.

**Figure 5:**
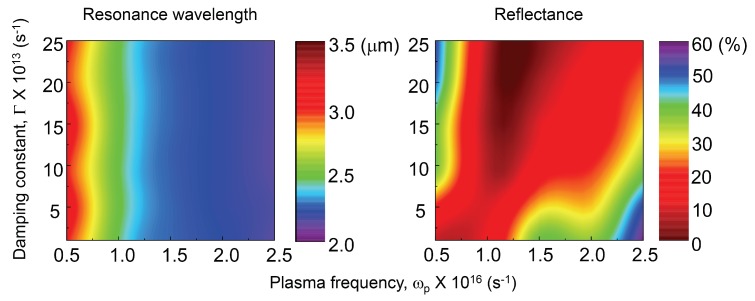
FDTD simulations with artificial Drude parameters that have $\omega_{p}$ from 0.5 to 2.5 × 10${}^{16}$ s${}^{-1}$, Γ from 1 to 5 × 10${}^{13}$ s${}^{-1}$ and $\varepsilon_{\infty }=-20.8$ respectively.

General tendencies of spectral response obtained from the series of FDTD simulations show that a large $\omega_{p}$ value defines a shorter resonance wavelength. The larger Γ defines lower reflectance at the resonance wavelength. The pairs $\omega_{p}$ and reflectivity *R*, Γ and resonance wavelength have no correlation with each other. These tendencies, however, are completely opposite to the experimental measurements of plasmon resonance from nano-disc structures [24,25,26]. To get insight for the underlying reasons of the experimentally observed tendencies, further FDTD simulations were performed using synthesised permittivities which have $\omega_{p}$ within the 0.5 to 2.5 × 10${}^{16}$ s${}^{-1}$, Γ changing from 1 to 25 × 10${}^{13}$ s${}^{-1}$, $\varepsilon_{\infty }=-20.8$ respectively. The bottom metal layer had default Palik’s data (Figure 5).

**Figure 6:**
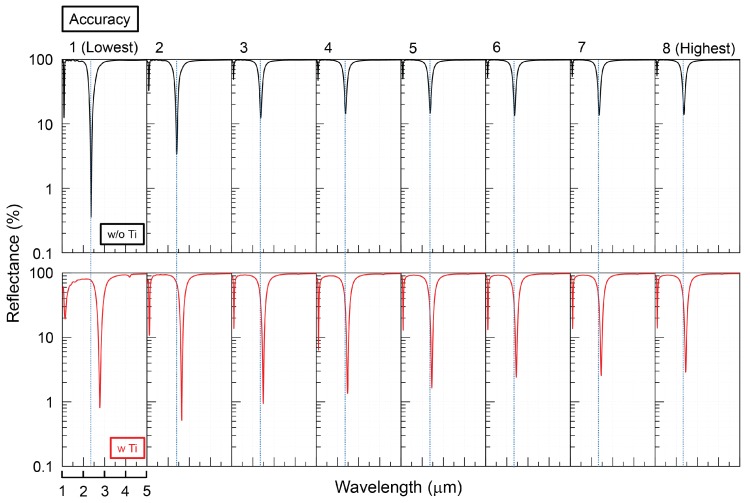
FDTD simulations with or without Ti adhesion layer (5 nm thickness) at several meshing accuracy levels (8 was the highest). The diameter of the disc is 300 nm, period is 1200 nm, SiO${}_{2}$ thickness is 50 nm; Palik’s data were used.

In the Lumerical FDTD solutions, automatic non-uniform meshing is essentially selected for simulation. Figure 6 shows the FDTD results with various meshing accuracy with/without Ti adhesion layer. When Ti adhesion layer is inserted, a ∼ 80 nm red shift and ∼ 10% of absorption increments were observed due to the electric permittivity of Ti and a 10 nm increase in separation between Au nanodisc and substrate. In the case of no Ti layer, the simulation results were stabilized after at least accuracy 3, however with the Ti layer the accuracy has to be higher than 6 ($∼10^{\times }$ more of simulation time and 3–6-fold increase in memory required).

The results of resonance wavelength and reflectance reproduce well the tendency of the experimental results. The shift of the resonance wavelength is related to the interaction between the top nano-disc and bottom Au layer. However, the plasma frequency is almost constant because gold has a fcc-crystal lattice and the free electron density is fixed by material. Therefore, the resonance wavelength obtained in experiments and simulations is the same. On the other hand, Γ is related to the electron damping at the grain boundaries, electron–electron and electron–hole scattering. Hence, it is related to the relaxation time of the plasmon resonance. At the plasmon resonance, a longer relaxation time is the reason for the higher Q-factor and strong electric field enhancement. However, in the absorptive metasurfaces, a large damping results in an increase of internal absorption due to ohmic losses in the metals. Also, a longer relaxation time might cause an increase in the influence of electron scattering on the plasmon resonance. Therefore, the tendencies observed in experiments are different to the typical plasmon resonance of nano-discs.

The values of Γ are affected by the structural quality of films and film deposition methods. The parameter $\Gamma =v_{F}/l$, where $v_{F}$ is the Fermi velocity of electron and *l* is its mean free path. For the electrons in nanoparticles where the boundaries add to the scattering when dimensions of the particles are smaller than *l*, the effective mean free path $1/l^{\prime }=1/l+a\left(v_{F}/R\right)$, where *R* is the size of nanoparticle and constant a≈1 [27]. Ab initio calculations based on electronic band diagram for gold provide $v_{F}=13.82\times 10^{5}$ m/s and l=37.7 nm, which can be considerably (more than 30%) different from the experimental or model based values [28]. In this study the effect of grain boundaries and film thickness when they are smaller than the free mean path of electron in bulk metal are reflected in the numerical and experimental data.

Absorption and reflectivity are strongly affected by the electron mean free path which is linked to the energy deposition. Usually, the effect of a radiative energy concentration via E-field enhancement due to the large value of the real part of refractive index is discussed. Here, we highlight the importance of the imaginary part of the refractive index in energy deposition for design of perfect absorbers (A→1 and R→0). It could be foreseen that optical properties (large Γ) of high entropy metal alloys (with more than 5 constituents) can provide high optical losses and be applied where strong absorbance is required. Black metals with R→0 and T→0 are made with thin <10 nm metal layer in IMI-metasurface [29] and their optical properties are defined by large losses.

Interestingly, the experimental data shows that the absorption value is close to the Mcpeak [18] or Olmon [19], which show the smallest damping constant. The experimental data would show high quality low loss metal film with Γ∼5×10${}^{13}$ s${}^{-1}$. The resonance wavelength determined from the experimental result is red-shifted, as would be expected from the plasma frequency $\omega_{p}∼$ 1.0×10${}^{16}$ s${}^{-1}$. For the best fitting of the experimental results by FDTD model, it is important to know precisely the electric losses, effective electron mass and free electron density. These parameters strongly depend on the film’s deposition conditions. Therefore, experimental determination of these parameters at the required spectral window would help to increase the accuracy of FDTD simulations for a given thicknesses of MIM metasurfaces.

### 3.3. The Effect of Adhesion Layer

Adhesion layers made of electrically high loss metals like Ti, Cr are frequently used. They can form an intermixing layer between metal and dielectric. Such a layer will have a coefficient of thermal expansion in between those for Si (with a SiO${}_{2}$ native oxide layer Si 2.6 × 10${}^{-6}$ K${}^{-1}$) and a metal: Au 14.2× 10${}^{-6}$ K${}^{-1}$, Ti 8.6 × 10${}^{-6}$ K${}^{-1}$, Cr 4.9 × 10${}^{-6}$ K${}^{-1}$. Due to the thin adhesion layer of 2–5 nm, a higher precision is required for the simulations as compared with the case without adhesion layer.

## 4. Conclusions and Outlook

A detailed comparison of experimental and numerical simulation was carried out for MIM metasurfaces to reveal peculiarities in how to tune the resonance wavelength and reflectance *R* of perfect absorbers at the mid-IR spectral range. Experimental data were collected from over 400 samples which were all made through the same deposition process and only differed by EBL-defined sizes of disks and period. All experimental results agreed well with FDTD simulations in the tendency of resonance wavelength and reflectance. FDTD results were strongly affected by the permittivity, especially the Γ value in the Drude model and thickness of the metal films in MIM metasurface. Therefore, it is important to carefully select the realistic permittivity model or to determine the permittivity of metals experimentally. In a series of experiments and simulations, we have revealed that metal with high ohmic loss demonstrates a higher potential to increase the absorption of metasurfaces. Experimentally, less than 5% reflection was achieved with MIM metasurfaces at specific spectral windows. This constitutes an engineering art to achieve A→1 in A+R+T=1.

The explored absorbance enhancement at the mid-IR spectral window is important for a number of bio-medical and environmental sensor applications, e.g., biological N_2_ fixation is the determining factor in CO_2_ uptake by plants from the atmosphere [30]. The atmospheric transmission window with T≈90% between 3 and 4 μm wavelengths can be utilised for radiative cooling. The Boltzmann temperature of 3.5 μm radiation is $T=h\nu /k_{B}=4.11\times 10^{3}$ K, defining the limit of the heat to work conversion in Carnot engine, where hν is the photon energy and $k_{B}$ is the Boltzmann constant. Structures and surfaces for high reflectivity or transmissivity at 1–4 μm IR wavelengths can be made by 3D laser polymerisation with direct writing or holographic recording [31,32,33]. Surfaces designed for enhanced absorption can be used as efficient radiators/emitters at the same wavelengths according to the Kirchhoff’s principle demonstrated experimentally at the IR spectral range [12]. Proposed metasurfaces might be useful for research in thermal generation of enhanced solar absorption, which promotes applications in water evaporation (solar steam generation) for solar central tower power plants and cleaner drinking water from sea water [34,35].
